# Clinical characteristics and mutation Spectrum of *NF1* in 12 Chinese families with orbital/periorbital plexiform Neurofibromatosis type 1

**DOI:** 10.1186/s12881-019-0877-9

**Published:** 2019-09-18

**Authors:** Peiwei Chai, Yingxiu Luo, Chuandi Zhou, Yefei Wang, Xianqun Fan, Renbing Jia

**Affiliations:** 1 Department of Ophthalmology, Ninth People’s Hospital, Shanghai JiaoTong University School of Medicine, No 639 Zhi Zao Ju Road, Shanghai, 200011 China; 2 Shanghai Key Laboratory of Orbital Diseases and Ocular Oncology, Shanghai, China

**Keywords:** Orbital neurofibromatosis, Orbital/periorbital plexiform neurofibroma (OPPN), *Neurofibromin 1 (NF1)* mutation

## Abstract

**Background:**

Orbital/periorbital plexiform neurofibroma (OPPN) can compromise physical appearance and visual function. However, the clinical characteristics and *NF1* mutation landscape in patients with heritable OPPN have not been reported.

**Methods:**

The medical charts of 26 Chinese patients with OPPN from 12 families were reviewed. Mutation analysis of the entire coding region and flanking splice sites of the *NF1* gene was performed using next-generation sequencing (NGS). Novel *NF1* mutations were confirmed by Sanger sequencing.

**Results:**

Compared to the parental generation, a significantly larger proportion of OPPN patients in the successive generation presented with earlier onset (*p* = 0.001), amblyopia (*p* = 0.034), motility disorders (*p* = 0.009) and bony orbital expansion (*p* = 0.019). Six novel *NF1* mutations were identified in 11 (91.67%) families, including 6 (42.9%) single-base substitutions, 4 (28.5%) splicing mutations, 3 (21.4%) frameshift deletions, and 1 (7.14%) intron mutation.

**Conclusions:**

The successive generation of OPPN patients presented with earlier onset and exhibited more severe ocular signs than did their parents or grandparents. Special attention should be paid to successive generations of OPPN patients. Considering that 6 mutations were novel, comprehensive *NF1* mutation analysis is required or necessary or proposed for genetic counselling.

**Electronic supplementary material:**

The online version of this article (10.1186/s12881-019-0877-9) contains supplementary material, which is available to authorized users.

## Background

Neurofibromatosis type 1 (NF1) is one of the most common autosomal dominant disorders [1], with an incidence of 1 per 2500 to 3000 individuals, independent of ethnicity and sex [2]. The most frequent clinical manifestations of NF1 patients include multiple benign neurofibromas, > 6 alterations of skin pigmentation (café au lait spots), iris (Lisch) nodules, skeletal abnormalities, learning disabilities, cardiovascular disorders and nervous system neoplasms, as well as malignant peripheral nerve sheath tumours [3]. Furthermore, the occurrence of serious complications increases with age. Therefore, NF1 is a fully penetrant neuro-cutaneous tumour-predisposing disorder with intractable complications.

The most disastrous subtype of NF1 is plexiform neurofibromatosis (PN) [4]. Approximately 30 to 56% of NF1 patients develop PNs, which may involve the eyelid, orbit and periorbital and facial structures [5, 6]. Orbital/periorbital PNs (OPPNs) can cause a dramatic change in physical appearance, such as proptosis, ptosis, and facial disfigurement, thus leading to decreased self-esteem, social embarrassment and mood disorders [7, 8]. In addition, OPPNs can cause various ocular complications, such as optic pathway gliomas, glaucoma, Lisch and choroidal nodules, ultimately resulting in loss of vision, especially during visual maturation [6] in children.

Neurofibromatosis type 1 is caused by a mutation in the *neurofibromin 1* (*NF1)* tumour-suppressor gene, which comprises 2350 kb and 60 exons on chromosome 17q11 [9]. The gene product neurofibromin (2818 amino acids) contains a domain with significant homology to Ras GTPase-activating proteins, and it regulates Ras activity [10]. Therefore, a lack of functional neurofibromin leads to dysregulated Ras signalling and tumourigenesis. PN consists of neoplastic Schwann cells, fibroblasts, perineural cells and mast cells. Neoplastic Schwann cells lack *NF1* gene expression and exhibit elevated levels of activated Ras. In turn, activated Ras initiates a cascade of signalling events, such as activation of Raf and mitogen-activated protein kinase, thus promoting cell proliferation. A previous study revealed numerous *NF1* gene mutations in lung cancer, glioblastoma, melanoma and cutaneous NF1 [6, 11, 12]; however, to our knowledge, the literature exploring the *NF1* mutations in OPPN patients is rather limited.

Here, we present in detail the clinical characteristics of Chinese patients with OPPN and investigate the *NF1* mutation spectrum.

## Methods

### Patients

A retrospective review was performed of consecutive patients diagnosed with OPPNs at Ninth People’s Hospital of Shanghai between January 2013 and June 2016. NF1 diagnosis was based on clinical features conforming to at least two of the following National Institutes of Health criteria [13]: six or more café au lait spots, axillary or inguinal freckling, two or more cutaneous neurofibromatosis, one plexiform NF, characteristic bony defects, optic glioma, two or more iris Lisch nodules, or a first-degree relative with NF1. Moreover, the probands of all families presented with plexiform NF in the orbital or periorbital region. The exclusion criteria were as follows: (1) a follow-up period < 6 months; and (2) incomplete data collection. We contacted all patients and their relatives who met the inclusion criteria and explained the purpose of the study; the participants were voluntary without any compensation. A total of 36 OPPN patients were included in this study. The OPPNs include 32 hereditary OPPNs (14 families) and 2 sporadic OPPNs. Of the 34 patients from 14 families who met the inclusion criteria, 4 were not reached, and 2 declined to participate for other reasons, such as time or geographic limitations. Of the 28 patients who agreed to return for the follow-up visit, 2 were excluded for incomplete data, leaving a final sample size of 26 patients. All 26 patients had familial OPPN.

### Data collection

Informed consent was obtained from all patients or their guardians at the follow-up visit. This study adhered to the tenets of the Declaration of Helsinki and was approved by the Shanghai Jiaotong University research ethics committee. The medical records were reviewed. Data collected included patient demographics, clinical characteristics, treatments and final outcomes at follow-up. The demographics consisted of age and sex. The clinical characteristics included the presence of amblyopia, motility disturbances, corneal changes, optic nerve disorders, ptosis, proptosis, canthal abnormalities, cafe au lait spots, facial descent, cheek deformities, bony orbital expansion and soft tissue expansion of eyelids. If the patients’ relative had received treatment elsewhere, his/her prior clinical details and pathological sections were retrieved for review. Regarding outcome measures, the duration from the initial diagnosis to the first recurrence was documented.

### Mutation identification

Genomic DNA was isolated from the peripheral blood leukocytes of the participants. We collected genomic DNA samples from at least two affected patients of each family (24 patients in 12 families and 2 unaffected volunteers were chosen as controls). The affected patients shared the same *NF1* mutation site, which was wild-type in the unaffected patients. These mutations were not present in 100 normal subjects or in the dbSNP database (http://www.ncbi.nlm.nih.gov/SNP), predicted to be not tolerated by SIFT (http://sift.bii.a-star.edu.sg/index.html) and to be a disease-causing variant by Mutation Taster (http://www.mutationtaster.org/). A panel of *NF1* gene exons, splicing sites and promoter regions was designed and sequenced following the instructions of Ion AmpliSeq™ Library Kit 2.0 (Thermo Fisher, USA). A targeted next-generation sequencing (NGS) approach, bioinformatics analyses, and Sanger sequencing were utilized. Library preparation, qualification, and NGS were conducted using the Illumina Hiseq2000 platform (Illumina, Inc., San Diego, CA, USA). Bioinformatics analyses, including read alignments and calculations of coverage and depth, were also carried out according to a previously described protocol [14]. The following 5 databases were used for annotation of all identified variants, including dbSNP137 (http://hgdownload.cse.ucsc.edu/goldenPath/hg19/database/snp137.txt.gz.), HapMap Project (ftp://ftp.ncbi.nlm.nih.gov/hapmap), 1000 Genomes Project (ftp://ftp.1000genomes.ebi.ac.uk/vol1/ftp), YH (http://yh.genomics.org.cn/), and Exome Variant Server (http://evs.gs. washington.edu/EVS/). *NF1* microarray data are listed in Additional file 1: Table S1.

### DNA extraction and sequencing

Genomic DNA was extracted from the patients’ peripheral blood leukocytes (51,206; QIAGEN, Hilden, Germany). The region of the mutated *NF1* genomic fragments was amplified by polymerase chain reaction (PCR), which was performed with 100 ng genomic DNA, 25 μL 2× GC-rich buffer, 8 μL of a dNTP mixture (2.5 mmol/L), 1 U LA Taq (Takara Biotechnology (Dalian) Co., Ltd), 2 μL (10 μmol/L) of each primer, and ddH_2_O to a final volume of 50 μL. The PCR amplification protocol was as follows: 95 °C for 5 min; 40 cycles at 95 °C for 30 s, 55 °C for 30 s, and 72 °C for 30 s; and a final elongation step at 72 °C for 3 min. The PCR products were then Sanger sequenced to produce a chromatogram. These primers used are listed in Additional file 2: Table S2.

### Statistical analysis

The data were analysed using SAS software (version 9.2, SAS Institute, Inc., Cary, NC). The frequency (percentage) and mean ± standard deviation are reported for categorical and continuous variables, respectively. Means and proportions were compared using Student’s t test and the chi-square test (or Fisher’s exact test, if appropriate), respectively. All tests were two-sided, and a *p* value < 0.05 was considered statistically significant. The Kaplan–Meier method was used to assess the cumulative recurrence rate.

## Results

### Clinical characteristics of heritable Chinese OPPN patients

**Fig. 1:**
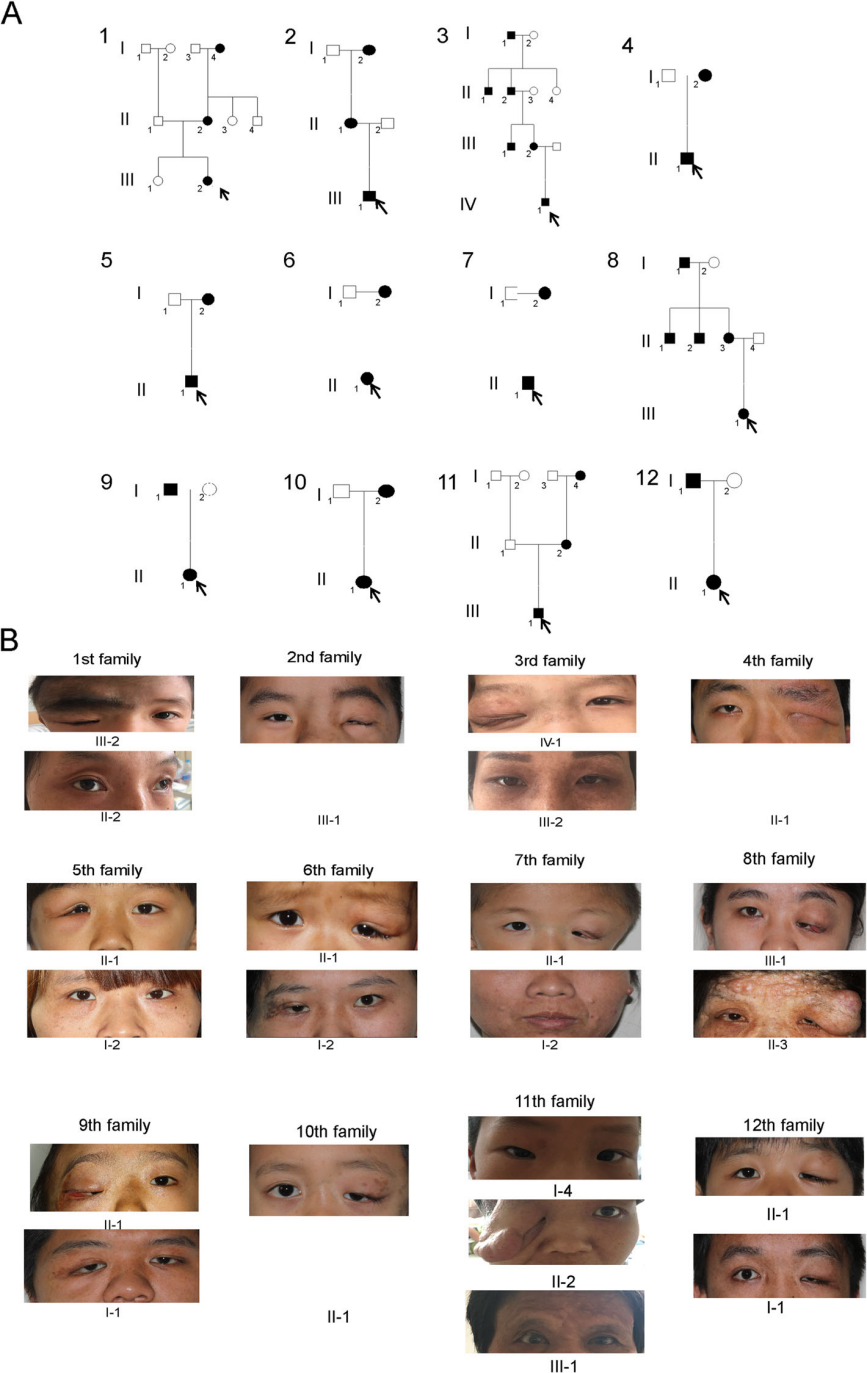
**a** Genogram of 12 Chinese orbital/periorbital plexiform neurofibromatosis (OPPN) families. The status of affected (black symbol) represents OPPN disease. **b** Various clinical appearances of Chinese OPPN patients

**Fig. 2:**
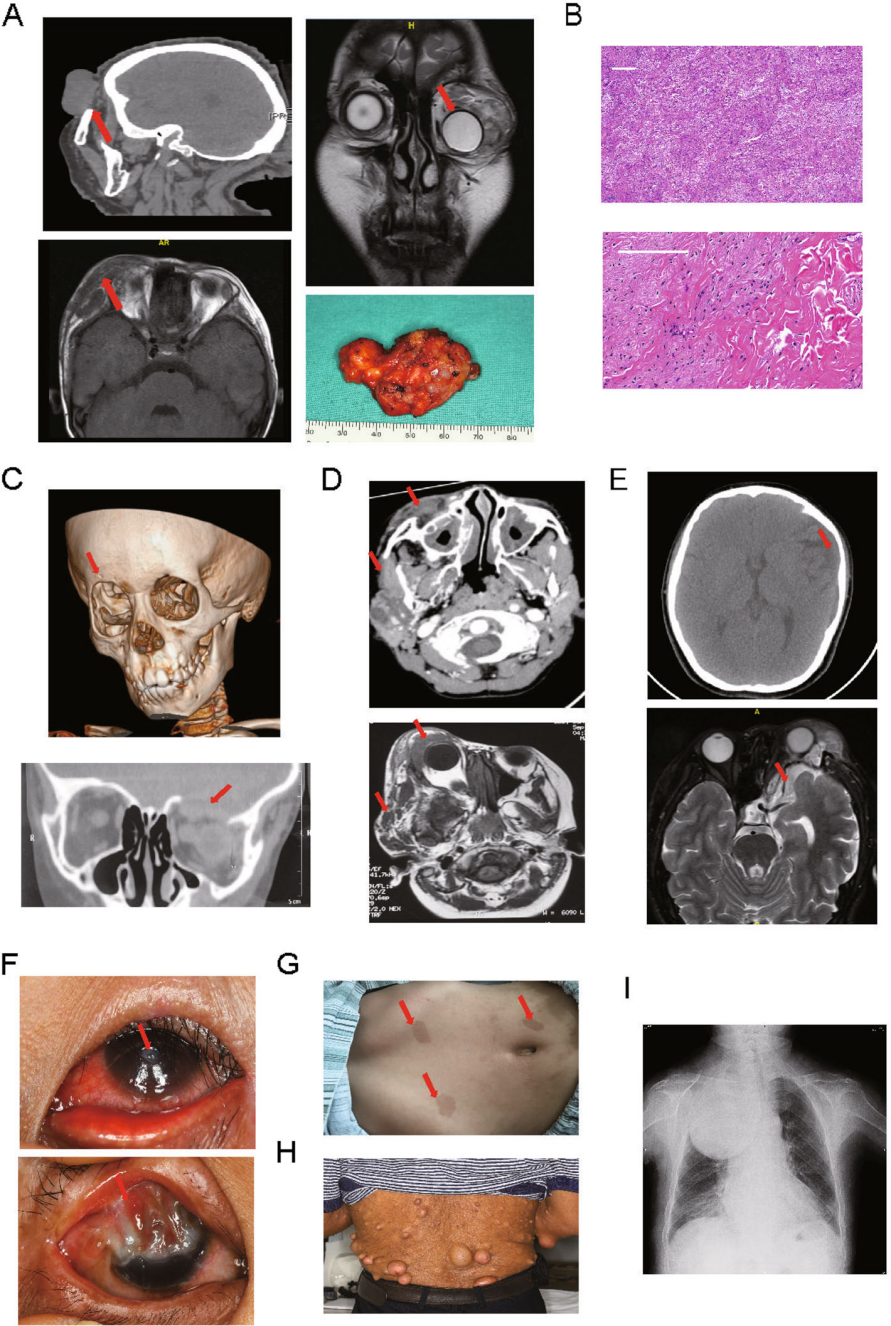
Clinical manifestations of orbital/periorbital plexiform neurofibromatosis (OPPN) patients. **a** OPPN located in the upper eyelid. Left: Computed tomography (CT) of infiltrative OPPN. Right upper: Magnetic resonance imaging (MRI) of infiltrative OPPN. The arrow indicates the orbital infiltration of OPPN and displacement of the orbital contents. Right below: General view of a dissected OPPN. **b** Pathological feature of OPPN: abundant collagen fibres and nerve sheaths. Scale bar: 40 μm. **c** CT images of OPPN resulting in orbital bone destruction. The arrow indicates the area of orbital osteoclasia. **d** CT images of facial infiltration of OPPN. The arrow indicates the infiltrated area of OPPN. **e** CT images of intracranial infiltration of OPPN. The arrow indicates the intracranial infiltration. **f** Upper: corneal ulceration. Below: Surgery-induced symblepharon. **g** Café au lait spots. **h** Coexisting extraocular OPPN. **i** X-ray images showing an enormous mass in the right chest of OPPN patients

A total of 26 eyes from 26 patients were recruited for this study. Among them, 9 patients (35%) were male and 17 (65%) female. The mean age was 26.9 ± 15.3 years, ranging from 6.0 years to 50.0 years. The mean follow-up period was 35.1 ± 19.6 months. Various clinical characteristics of Chinese patients with OPPN are displayed in Fig. 1. The most commonly involved area is the upper eyelid (Fig. 2a). Histologically, the nodules consisted of abundant collagen fibre bundles, nerve sheaths and Schwann cells (Fig. 2b). Moreover, these OPPN patients often exhibited orbital expansion (Fig. 2c), facial osteoclasia (Fig. 2d), intracranial invasion (Fig. 2e), corneal ulcer and symblepharon (Fig. 2f). Systematic manifestations, such as café au lait spots (Fig. 2g) and coexisting extraocular NFs nodules (Fig. 2 h, i), were also found.

Demographic and clinical characteristics were compared between the patients and their previous generation, and the results are summarized in Table 1. Most patients exhibited OPPN in the same laterality as their parents and/or grandparents. Of note, significantly earlier onset (*p* = 0.001) and more severe symptoms were observed in successive generations. Additionally, compared to their ancestors, a larger proportion of patients displayed amblyopia (*p* = 0.034), motility disorders (*p* = 0.009) and bony orbital expansion (*p* = 0.019). However, no statistical significance was found regarding the following: sex; the presence of ptosis; proptosis; cafe au lait spots; facial descent; cheek deformities; soft tissue expansion of the eyelids or corneal or optic nerve; or canthal abnormalities. Among the 12 patients who underwent reconstruction surgery at our hospital, 10 (83.3%) experienced recurrence. Based on Kaplan-Meier survival estimates, the 1-year recurrence rate was 83.3%, and the median duration to the initial recurrence was 7.0 months (95% confidence interval: 5.30–8.70 months).

**Table 1:** Comparisons of demographic and clinical characteristics between the patients and affected members of the previous generation

	Total (*n* = 26)	Patient (*n* = 12)	Parents and/or grandparents (*n* = 14)	*p*
Sex				0.127
Male	9(35%)	6(50%)	3(21%)	
Female	17(65%)	6(50%)	11(79%)	
Age of onset	16.0 ± 10.1	9.5 ± 4.5	21.6 ± 10.4	0.001*
Amblyopia				0.034*
Present	16(62%)	10(83%)	6(43%)	
Absent	10(38%)	2(17%)	8(57%)	
Motility disorders				0.009*
Present	17(65%)	11(92%)	6(43%)	
Absent	9(35%)	1(8%)	8(57%)	
Corneal abnormalities				0.490
Present	5(19%)	3(25%)	2(14%)	
Absent	21(81%)	9(75%)	12(86%)	
Optic nerve abnormalities				0.449
Present	3(12%)	2(17%)	1(7%)	
Absent	23(88%)	10(83%)	13(83%)	
Ptosis				0.345
Present	25(96%)	12(100%)	13(93%)	
Absent	1(4%)	0(0%)	1(7%)	
Proptosis				0.127
Present	9(35%)	6(50%)	3(21%)	
Absent	17(65%)	6(50%)	11(79%)	
Canthal abnormalities				0.356
Present	22(85%)	11(92%)	11(79%)	
Absent	4(15%)	1(8%)	3(21%)	
Café au lait spots				1.000
Present	26(100%)	12(100%)	14(100%)	
Absent	0(0%)	0(0%)	0(0%)	
Facial descent				0.126
Present	11(44%)	7(58%)	4(29%)	
Absent	15(56%)	5(42%)	10(71%)	
Cheek deformities				0.126
Present	11(44%)	7(58%)	4(28%)	
Absent	15(56%)	5(42%)	10(72%)	
Bony orbital expansion				0.019*
Present	9(35%)	7(58%)	2(14%)	
Absent	17(65%)	5(42%)	12(86%)	
Soft tissue expansion of eyelids				0.173
Present	25(96%)	12(100%)	12(86%)	
Absent	1(4%)	0(0%)	2(14%)	

*Statistically significant

### Novel *NF1* mutations in Chinese OPPN families

**Fig. 3:**
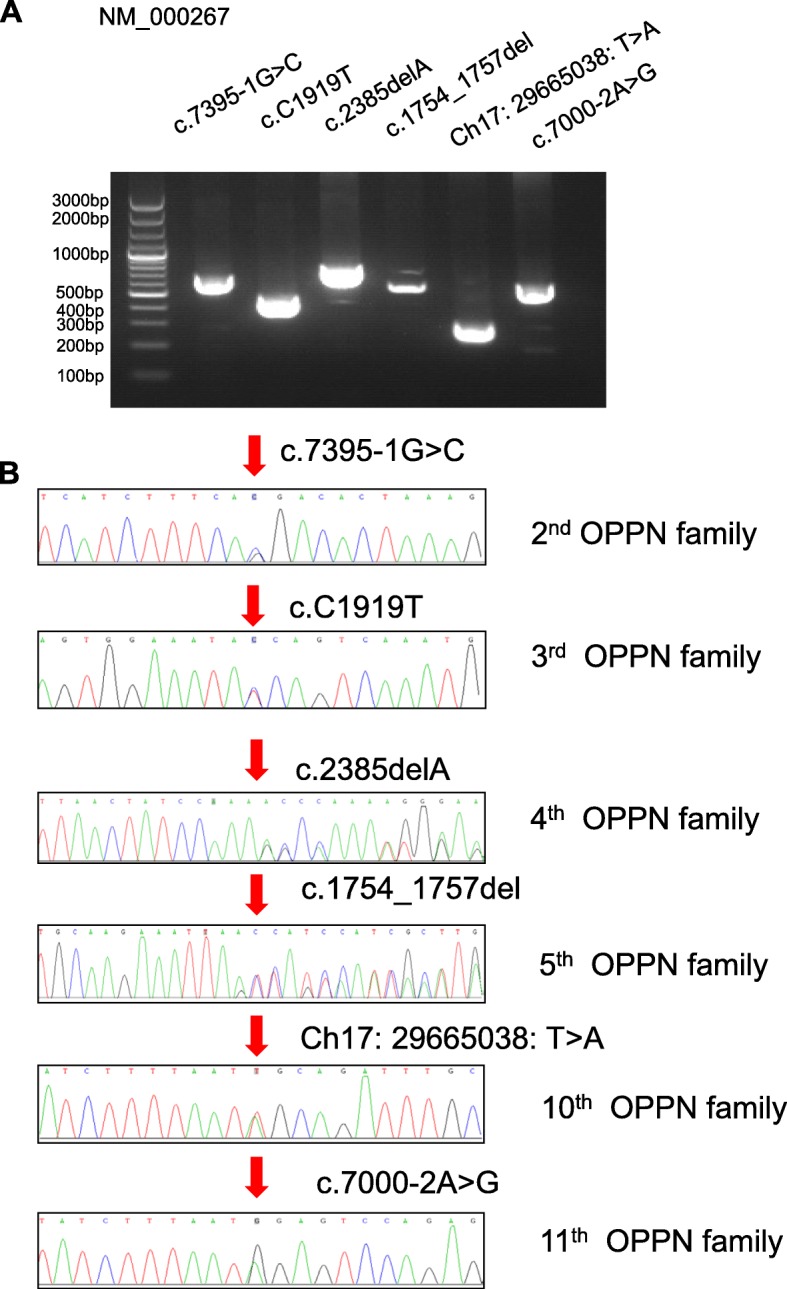
PCR gel images and Sanger chromatogram indicating novel *NF1* mutations. **a** PCR gel images of each mutated *NF1* genomic fragment. **b** Sanger chromatogram of each novel *NF1* mutation

Mutation analysis revealed a wide spectrum of *NF1* mutations in this cohort (Table 2), with fourteen different mutations identified. Eleven (91.7%) OPPN families presented *NF1* mutations, and 6 identified mutations were novel. Nine (64.3%) of the fourteen identified mutations were distributed in exons, three (21.4%) in splicing sites and one (7%) in an intron. Among exon mutations, three (33.3%) nonsense mutations causing premature termination of neurofibromin were detected; three frameshift mutations and three missense mutations were also discovered. These mutations were evenly distributed from exon 12 to intron 49 of the gene. Additionally, no common mutations were detected across the families, suggesting the absence of mutational hot spots. The regions of *NF1* genomic DNA mutation sited were amplified by PCR (Fig. 3a), and a Sanger chromatogram confirmed novel mutations (Fig. 3b).

**Table 2:** Mutation spectrum of *neurofibromin 1* in Chinese orbital-periorbital neurofibromatosis patients

Family number	NF1 mutation	Region	Mutation type	Mutation site	Protein	Novel/ previously described
1	Yes	Exon 49	Frameshift deletion	NM_000267: exon49: c.7385_7394del: p.P2462fs, NM_001042492: exon50: c.7448_7457del: p.P2483fs	P2462fs	previously described
2	Yes	Splicing	/	NM_001042492: exon51: c.7458-1G > C, NM_000267: exon50: c.7395-1G > C	/	Novel
3	Yes	Exon 17	Missense SNV	NM_000267: exon17:c.C1919T: p.T640I, NM_001042492: exon17:c.C1919T: p.T640I	p.T640I	Novel
		Exon 18	Stop gain	NM_000267: exon18:c.C2041T: p.R681X, NM_001042492: exon18:c.C2041T: p. R681X	p.R681X	previously described
4	Yes	Exon 20	Frameshift deletion	NM_000267: exon20: c.2385delA: p.P795fs, NM_001042492: exon20: c.2385delA:p.P795fs	p.P795fs	Novel
5	Yes	Exon 16	Frameshift deletion	NM_000267: exon16: c.1754_1757del: p.L585 fs, NM_001042492: exon16: c.1754_1757del:p.L585 fs	p.L585 fs	Novel
		Exon 17	Missense SNV	NM_000267: exon17: c. A1933G: p.M645 V, NM_001042492: exon17:c. A1933G:p.M645 V	p.M645 V	previously described
6	Yes	Splicing	/	NM_001042492: exon36: c.4725-1G > A, NM_000267: exon35: c.4662-1G > A	/	previously described
7	Yes	Exon 21	Missense SNV	NM_000267: exon21: c. A2683G: p.M895 V, NM_001042492:exon21:c.A2683G: p.M895 V	p.M895 V	previously described
		Splicing	/	NM_001042492: exon16: c.1845 + 1G > A, NM_000267: exon16: c.1845 + 1G > A	/	previously described
8	No	/	/	/	/	/
9	Yes	Exon 12	Stop gain	NM_000267: exon34:c.C4537T: p.R1513X, NM_001042492: exon35:c.C4600T: p.R1534X	p.R440X	previously described
10	Yes	Intron	/	NF1:Ch17: 29665038: T > A	/	Novel
11	Yes	Splicing	/	NM_001042492: exon48: c.7063-2A > G, NM_000267: exon47: c.7000-2A > G	/	Novel
12	Yes	Exon 34	Stop gain	NM_000267: exon34:c.C4537T: p.R1513X, NM_001042492: exon35:c.C4600T: p.R1534X	p.R1513X	previously described

## Discussion

OPPN is a severe form of NF with a short, non-progressive period [6]. This study constitutes the largest cohort of heritable OPPNs. The successive generation presented earlier onset and exhibited more severe ocular signs, including amblyopia, motility disorders and bony orbital expansion, when compared with their parents or grandparents. In general, earlier onset can result from an increased awareness of the disease in a family. Each Chinese family with OPPN in our study shared one common *NF1* mutation; however, the underlying reason for the aggravated symptoms in the successive generation remains unclear. Notably, no common mutations were detected across the OPPN families, indicating that there were no mutational hot spots. Additionally, no obvious genotype-phenotype correlation was observed in the OPPN patients.

To date, more than 2000 different *NF1* mutations have been identified in Human Gene Mutation Database [15]. The *NF1* gene has one of the highest mutation rates, and different populations have different sets of recurrent exonic mutations [6, 16, 17]. Our study detected 6 novel mutations in 26 OPPN patients among 12 families. Of note, an intron mutation (NF1:Ch17: 29665038: T > A) was identified in the patients of the 10th family. Although two patients shared this mutation and this mutation was not present in unaffected individuals, we cannot confirm whether this intronic mutation is related to the pathogenesis of OPPN. We performed a literature review as well as a comparison of *NF1* mutations in Chinese and patients from other parts of the world [16, 17]. The results indicated that the missense mutation rate in Italian patients (58.9%) was significantly higher (all *p* = 0.01) than in Korean (38.5%) and Chinese (21.4%) patients (Table 3). To date, there has been little evidence with regard to the genotype-phenotype correlation in *NF1*. Although OPPN patients in one family share a common *NF1* mutation, symptoms can vary greatly. This might be due to differences in age, sex and other epigenetic factors [18]. For example, a family-based association study revealed a strong association between the lncRNA *ANRIL* and the number of plexiform neurofibromas [19].

**Table 3:** Comparisons of *NF1* mutation spectra between Chinese and patients from other parts of the world

	Chinese patients (*n* = 14)	Korean patients (*n* = 52)	Italian patients (*n* = 73)	p1	p2
Novelty				0.395	0.591
Reported	8(57.1)	36(69.2)	36(49.3)		
Novel	6(42.8)	16(30.8)	37(50.7)		
Single-base mutation	6(42.9)	30(57.7)	49(67.1)	0.322	0.085
Nonsense	3(21.4)	10(19.2)	6(8.2)	0.854	0.137
Missense	3(21.4)	20(38.5)	43(58.9)	0.235	0.010*
Splicing mutation	4(28.6)	8(15.4)	18(24.7)	0.256	0.758
Intron	1(7.14)	/	/	NA	NA
Frameshift deletion	3(21.4)	8(15.4)	18(24.7)	0.590	0.796
Insertion	0(0)	3(5.8)	1(1.4)	0.358	0.660

Data are presented as n(%)

*Statistically significant; p1: Chinese versus Korean patients [17]; p2: Chinese versus Italian patients [16]

Among NF1 patients, 35–60% of cases are familial and others sporadic [16, 17]. We initially enrolled 34 patients, including 32 (94.12%) familial and 2 (5.89%) sporadic cases. However, two sporadic cases were excluded due to loss of contact; thus, all of the OPPN cases were hereditary. The percentage of familial NF in out study was much higher than that previously reported, which could be partially explained by the following reasons: 1) increased awareness of this disease for hereditary cases, 2) selection bias with a sample size of 26 OPPNs and 3) OPPNs presented with higher genetic predisposition than did other types of NFs.

This study should be regarded as an initial exploration of the clinical characteristics and genetic patterns of Chinese patients with OPPN. Caution should be taken when interpreting the findings due to the following limitations. The sample size was small, though the low incidence of this disease makes it difficult to recruit a larger cohort of patients. In addition, this study was conducted in a single tertiary institution, and all participants were of Chinese Han ethnicity. All these factors may cause selection bias, and our findings may not be generalizable to all Chinese OPPN patients. Thus, we will continue to seek data sets from other medical centres for future validation studies. Nevertheless, we attempted to circumvent these limitations as follows: our study is the largest cohort of Chinese patients with familial OPPN to date, and this study involved a single surgeon and used objective statistical methods, which help to minimize selection and confounding biases.

## Conclusion

Earlier onset and more severe symptoms were displayed in successive generations of patients with heritable OPPN. Special attention should be paid to the successive generations of OPPN patients. Considering that some of the detected mutations were novel, comprehensive *NF1* mutation analysis is valuable for genetic counselling. To our knowledge, this is the first study to provide detailed clinical characteristics and the *NF1* mutation spectrum for Chinese patients with heritable OPPN. Therefore, a larger cohort from multiple clinical centres is required to fully validate our findings.

## Additional files

Additional file 1: Raw NF1 microarray sequencing data of OPPNs. (XLS 139 kb)

Additional file 2: Primers used in the study. (DOCX 12 kb)

## Data Availability

The raw sequencing data have been uploaded as supplementary files. The datasets analysed during the current study are available in the ClinVAR database (https://www.ncbi.nlm.nih.gov/clinvar/, accession number: VCV000638015, VCV000635821, VCV000635820, VCV000635819, VCV000635818 and VCV000186215).

## Abbreviations

CT: Computational tomography
ELISA: Enzyme-linked immunosorbent assay
MRI: Magnetic resonance imaging
NF: Neurofibromatosis
NF1: Neurofibromatosis type 1
OPPN: Orbital/periorbital PN
PN: Plexiform neurofibromatosis
