# Supplementary material for: Clinical characteristics and mutation Spectrum of NF1 in 12 Chinese families with orbital/periorbital plexiform Neurofibromatosis type 1
Source: BMC Med Genet. 2019 Sep 18;20:158. doi: 10.1186/s12881-019-0877-9 (PMC6749707; doi:10.1186/s12881-019-0877-9)
Supplement: Supplementary file 2 — Primers used in the study. (DOCX 12 kb) [file 12881_2019_877_MOESM2_ESM.docx]

**Supplementary Table 2**

c.7395-1G>C:

NF1-51-F 5' GTGCAAGATACTATGGCAAGAAAG 3'

NF1-51-R 5' GGCAAAACAAAATAAGGAGAGTC 3'

c.C1919T

NF1-17-F 5' CTCCTTCAAGTTGGGGCATAG 3'

NF1-17-R 5' CCAAAGTTCCACAAAGAGCAGTA 3'

c.2385delA

NF1-19-20-F 5' CTTCCTACTCCTTTTGGGTGG 3'

NF1-19-20-R 5' TGGCTACTGGTACTTGCGATG 3'

c.1754_1757del

NF1-16-F 5' TAGGGGGCTTATAGATAAATACTTG 3'

NF1-16-R 5' TCATCAACAGTGTGGTTCTAAGG 3'

Ch17: 29665038: T>A

Ch17: 29665038-F 5' CAGAACTAGCTCAAAGGTATGTCC 3'

Ch17: 29665038-R 5' AAGTGAGGGCGGAACAGGTA 3'

c.7000-2A>G

NF1-48-F 5' GATTTCATCTTCCACCATCTTCT 3'

NF1-48-R 5' CCTCAAACTCCTAGCCTCAAG 3
